# Development and Content Validation of a Comprehensive Health Literacy Survey Instrument for Use in Individuals with Asthma during the COVID-19 Pandemic

**DOI:** 10.3390/ijerph19041923

**Published:** 2022-02-09

**Authors:** Claudia Hasenpusch, Uwe Matterne, Christina Tischer, Ilona Hrudey, Christian Apfelbacher

**Affiliations:** 1 Institute of Social Medicine and Health Systems Research, Medical Faculty, Otto Von Guericke University, 39120 Magdeburg, Germany; uwe.matterne@med.ovgu.de (U.M.); ilona.hrudey@med.ovgu.de (I.H.); christian.apfelbacher@med.ovgu.de (C.A.); 2 Institute for Clinical Epidemiology and Biometry, University of Wuerzburg, 97080 Wuerzburg, Germany; Tischer_C@ukw.de; 3 State Institute of Health, Bavarian Health and Food Safety Authority, 91058 Erlangen, Germany

**Keywords:** SARS-CoV-2, COVID-19, asthma, survey instrument, questionnaire development, health literacy

## Abstract

Individuals with chronic conditions have been faced with many additional challenges during the COVID-19 pandemic. Individual health literacy (HL) as the ability to access, understand, evaluate, and apply pandemic-related information has thus become ever more important in these populations. The purpose of this study was to develop and content-validate a comprehensive HL survey instrument for people with asthma based on an integrated framework, and on previous surveys and other instruments for use in the general population and vulnerable groups. Beside HL, assumed determinants, mediators, and health outcomes were embraced in the framework. A mixed-method design was used. A comprehensive examination of the available literature yielded an initial pool of 398 single items within 20 categories. Based on content validity indices (CVI) of expert ratings (n = 11) and the content analysis of cognitive interviews with participants (n = 9), the item pool was reduced, and individual items/scales refined or modified. The instrument showed appropriate comprehensibility (98.0%), was judged relevant, and had an acceptable CVI at scale level (S-CVI/Ave = 0.91). The final version comprises 14 categories measured by 38 questions consisting of 116 single items. In terms of content, the instrument appears a valid representation of behavioural and psychosocial constructs pertaining to a broad HL understanding and relevant to individuals with asthma during the COVID-19 pandemic. Regular monitoring of these behavioural and psychosocial constructs during the course of the pandemic can help identify needs as well as changes during the course of the pandemic, which is particularly important in chronic disease populations.

## 1. Introduction

In the context of the infectious coronavirus disease (COVID-19), which is caused by severe acute respiratory syndrome-coronavirus-2 (SARS-CoV-2), health literacy (HL) and other constructs such as resilience and availability of social support are becoming increasingly important factors that help in coping with the crisis [1,2]. HL is the ability to find, understand, evaluate, and apply relevant health information that helps a person make health-related judgments and decisions in their everyday life [3]. HL can also be understood as a psychosocial resource that contributes to maintaining quality of life and promoting health in the long term [3]. HL is considered as a decisive factor in people’s ability to adhere to infection control measures [4]. It helps individuals to access COVID-19-relevant health information, recognise trustworthy sources, understand recommendations for preventive as well as protective measures, and integrate them into personal behaviours and actions [4,5]. It has been argued that low pandemic-related HL can be considered a risk factor that is associated with significantly lower informedness and reduced occurrence of preventive behaviours [6,7]. Further, it is assumed that HL is an indispensable psychosocial determinant of health in the context of the pandemic [1,2].

The findings of the Health Literacy Survey Germany 2 (HLS-GER-2) from 2020 show that more individuals with chronic diseases (62.3%) have limited HL compared to the general population (58.8%) [8]. Similar trends were observed in comparable surveys such as the HLS-EU [9], the Health Literacy Survey Germany 1 (HLS-GER 1) [10], the Health Literacy Survey Germany 1’ (HLS-GER 1’) [10], and the study “German Health Update study” (GEDA) [11]. From 2014 to 2020, the proportion of persons with inadequate HL levels among chronically ill persons has increased (2014: 16.8% vs. 2020: 26.8%) [10]. 

Asthma, a chronic inflammatory respiratory disease, affecting children and adults alike, is one of the most common non-communicable diseases worldwide. Its prevalence is still expected to rise, particularly in developing countries [12]. Primary symptoms comprise variable airflow obstruction, shortness of breath, cough, wheezing, and chest tightness. Other recurrent symptoms can occur, for instance, insomnia, daytime fatigue, decreased performance, physical limitations, and absenteeism from school or work [13]. As such, it places a high burden on individuals, families, and health systems [14,15,16]. The World Health Organization (WHO) classifies asthma as a serious global health problem with particular public health relevance [13].

Viral infections, particularly by rhinoviruses, are one of the most common triggers of asthma exacerbation [17,18,19]. In the context of the COVID-19 pandemic, a broad spectrum of clinical studies reported that the likelihood of severe as well as lethal COVID-19 disease courses is significantly higher in individuals with pre-existing chronic conditions compared to other populations [20,21]. Whether the likelihood of infection or a severe COVID-19 disease course is increased in asthma remains a subject of controversy. Synthesised evidence suggests that asthma does not increase the risk of COVID-19-related infection, mortality, or a severe cause or hospitalisation [22]. A systematic review concluded that asthma was not associated with negative COVID-19-related health outcomes [23,24]. However, another review concluded that high quality evidence is needed in order to answer that question [25]. 

Due to constant emergence of new evidence and high amounts of pandemic-related information, people with asthma have been facing various new challenges since the onset of the COVID-19 pandemic. In general, the COVID-19 pandemic poses psychosocial, economic, and political challenges that directly and indirectly impact population health and well-being [26,27,28,29]. Individuals with pre-existing conditions often perceive the infection control measures associated with containment (e.g., social distancing) as more onerous than the general population [26]. While worries and anxiety are generally higher in individuals with chronic diseases [7,30], there have also been reports of a significant increase in newly diagnosed anxiety disorders in individuals with asthma in 2020 compared to 2019 [30]. The prevalence of anxiety and affective disorder in individuals with asthma is generally higher than in the general population and these conditions are often associated with poor asthma control and low medication adherence [29,31]. A key challenge for individuals with asthma is to distinguish asthma symptoms from COVID-19 symptoms. In addition, anxiety, uncertainty, and other psychological effects can encumber effective asthma control and medication management (e.g., inhaled corticosteroid therapy) [26,29]. The pandemic may exacerbate existing impairments and give rise to new mental and other health problems (e.g., insomnia, depression), which in turn may have a negative impact on objective and perceived disease status [28,29]. 

Further, overtaxing amounts of information that have circulated during the pandemic are trying both the general population and individuals with pre-existing conditions [1,6]. There has been a significant increase in the dissemination of valid as well as non-evidence-based, inaccurate, or deliberately false pandemic-related information since the onset of the COVID-19 pandemic. The WHO (2020) declared this circumstance as an information epidemic, the so called ‘infodemic’ [6,32]. Inconsistent and unclear health information can be considered a stress factor for individuals with chronic lung disease and other populations, as it leads to confusion and uncertainty [6,26,33]. This may diminish one’s ability to critically evaluate the trustworthiness of information, and in turn affect the ability to make informed health-related decisions and the likelihood of engaging in preventive behaviours [1,7,34]. In relation to the chronical disease asthma, there is an association between low HL and negative outcomes in terms of disease coping, management, treatment adherence, and utilisation of health care services [35,36,37]. This association may be more pronounced during the COVID-19 pandemic [35,38]. Consequently, there is a need to know more about the levels of HL in populations with pre-existing conditions [6]. In terms of the assessment of COVID-19-related HL, synthesised evidence shows that previous studies predominantly focus on the general population [39]. Additionally, it is also important to identify other factors related to the pandemic, such as risk perception, preventive and information-seeking behaviour, or affective factors in the context of HL [6,35]. In light of the particular challenges individuals with asthma face during the COVID-19 pandemic, it was the aim of the present study to develop and content-validate a comprehensive survey instrument for the assessment of HL in individuals with asthma in the context of the COVID-19 pandemic.

## 2. Materials and Methods

**Figure 1:**
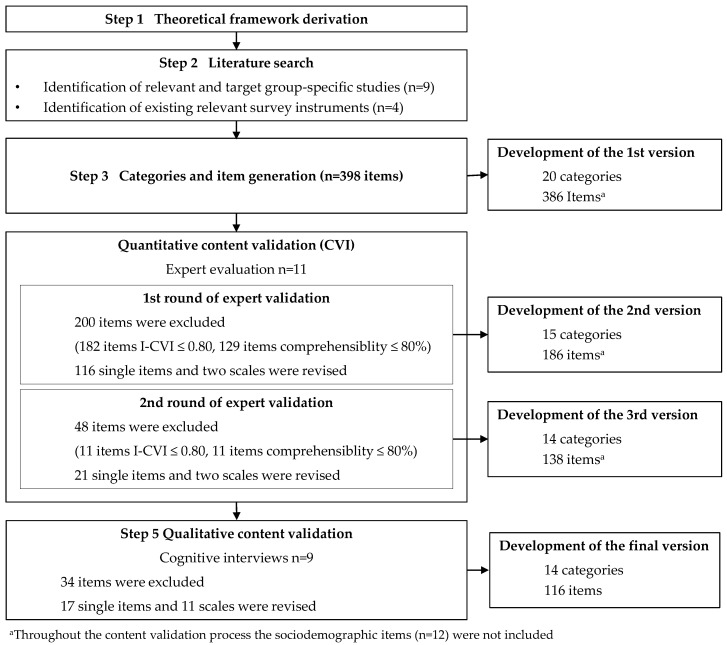
Study approach for developing the instrument.

A multi-stage approach was utilised to develop and content-validate a target group-specific survey instrument: (1) theoretical framework derivation, (2) literature search, (3) categories and item pool generation, assessment of content validity (4) by content validity indices (CVI) through expert ratings, and (5) cognitive interviews with participants with asthma (Figure 1). The latter used the Committee for Consensus-based Standards for the Selection of Health Measurement Instruments (COSMIN) recommendations referring to the three aspects of an instrument’s content validity—relevance, comprehensibility, and comprehensiveness [40]. The study was approved by the Ethics Committee of the Medical Faculty of the Otto von Guericke University Magdeburg (111/202, 31 July 2020). 

### 2.1. Theoretical Framework

**Figure 2:**
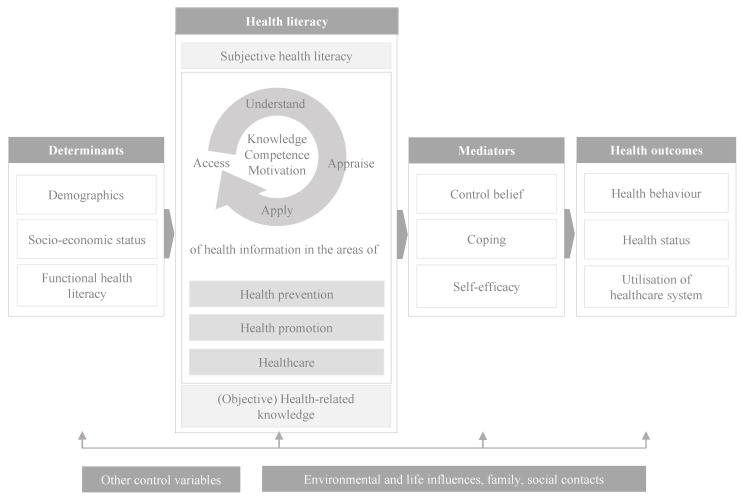
Conceptual framework adopted from Sørensen et al. [3], Schaeffer et al. [41], and Messer et al. [42].

We used a theoretical HL framework as a heuristic guide for the development of the survey. This framework was derived from work by Sørensen et al. [3], Schaeffer et al. [41], and Messer et al. [42]. It distinguishes between determinants of HL (e.g., sociodemographic information), HL, mediators (e.g., self-efficacy, coping) between HL and health outcomes (e.g., health behaviour, health status) and other control variables (e.g., risk perceptions), and environmental and life determinants (e.g., family or social contacts) (Figure 2). The structure and content of the survey instrument was informed by ‘WHO’s Survey Tool and Guidance—Rapid, simple, flexible behavioural insights on COVID-19′ [43] as it provides practical operationalisation guidelines, i.e., how the respective constructs can be measured.

### 2.2. Literature Search

We carried out an additional literature search (PubMed, EBSCOhost, MEDLINE^®^, PsychINFO^®^, SocINDEX^®^, Google Scholar^®^) to identify existing instruments relevant to our research interest. The search took place over a period from July 2020 to February 2021 in order to capture the currency of the dynamic evidence situation. All identified search results were imported into Citavi for further processing. The literature review was performed by two reviewers (CH, IH). Studies were included if they met the following criteria: (1) published in English or German between 1990 and 2021, (2) previously validated or frequently used HL instruments for use in the general population or in individuals with chronic diseases, (3) COVID-19-related HL. Additional relevant literature was identified by checking the reference lists. Key search terms were ‘health literacy’, ‘health’, ‘competence’, ‘literacy’, ‘knowledge’, ‘attitude’, ‘skills’, ‘self-efficacy’, ‘coronavirus’, ‘SARS-CoV-2’, ‘COVID-19′, chronic* conditions’, ‘long term condition’, ‘chronic disease’, ‘asthma’. The literature search was based on the approach suggested by Muka et al. [44]. 

### 2.3. Assessment of Content Validity

An expert panel (asthma practitioners and researchers) evaluated the content by completing a semi-structured questionnaire with open-ended questions (n = 5) and standardised items (n = 398 items). Two criteria of content validity were measured: relevance (‘How relevant do you consider this item to be?’) was assessed on a 4-point Likert scale (1 = ‘not relevant ‘, 2 = ‘somewhat relevant’, 3 = ’quite relevant’, and 4 = ‘highly relevant’) [45] and comprehensibility (‘Is this item clearly and comprehensibly formulated?’) was assessed by 1 = ‘unclear/ incomprehensible’ and 2 = ‘clear/ comprehensible’ [46]. Experts were also asked to provide comments on items they had particular expert knowledge about (qualitative assessment). Quantitative data were analysed by computing content validity indices (CVI at item level (I-CVI) and at scale level of the individual categories as well as over the entire instrument (S-CVI/Ave)). Items with I-CVI values of ≤0.80 were revised or removed by considering the written comments of the experts [45]. Based on the assumption that insufficient comprehensibility negatively influences the reliability of the instrument, questions with an agreement of less than 80% despite appropriate I-CVI values of 1.00, were modified [47]. The results of the first round of content validity assessment led to a second draft. Due to the findings of the first CVI assessment, a second round of expert rating took place after removal of incomprehensible and irrelevant items. This revision of items led to the third draft version. The expert rating lasted from August to October 2020. 

Next, cognitive interviews were carried out with members of the intended target group (adult participants with asthma). From August to November 2020, we recruited participants through social media channels, outpatient and inpatient hospitals, self-help groups, and snowballing. Participants received the preliminary version of the instrument by either mail or email (depending on stated preference). A semi-structured interview guideline was developed. We used the ‘Think Aloud’ technique, to identify potential comprehension problems and whether content was missing (comprehensiveness) [48]. Depending on the responses of the participants, two approaches were used (Concurrent or Retrospective Think Aloud). For example, when participants expressed difficulties to verbalise their experiences and thoughts (Concurrent Think Aloud), the Retrospective Think Aloud method was used. In addition, the probing technique was also used to elaborate [48]. Interviews lasted on average 79 min (range: 60 to 99 min). The interviews took place by telephone between the 2nd and 25th of November 2020 and were recorded and selectively transcribed, i.e., only relevant statements were transcribed based on the objective of identifying comprehension problems. Data were structured according to Prüfer and Rexroth (2005) [48] and content-analysed [49] by one coder (CH) using MAXQDA 2020(VERBI–Software. Consult. Sozialforschung. GmbH, Berlin, Germany). One third of the coded transcripts (n = 3) was reviewed independently by another reviewer (IH) to verify the accuracy of the coding data [50,51]. Disagreements were discussed (consensual coding) [51,52]. Based on the results of the cognitive interviews, questions that were difficult to understand, misleading, redundant, or incomplete were revised or removed and led to the final version of the instrument. Each revision was carried out by one member of the study team (CH). The quality of the development process was guided by the COSMIN Risk of Bias Checklist [40].

## 3. Results

### 3.1. Item Generation

Three hundred and ninety-eight single items (29 scales) pertaining to 20 categories suggested by ‘WHO’s Survey Tool and Guidance’ [43] as well as the WHO guideline-based COSMO Study Germany [53] were identified and formed the first draft of the instrument. Three additional categories were identified from the literature search. The categories pertain to: (1) sociodemographic data, (2) personal experiences with the COVID-19 pandemic or an infection, (3) subjective (COVID-19-related health information management) and objective health literacy (COVID-19 knowledge), (4) risk perceptions (vulnerability and severity), (5) preparedness and perceived self-efficacy, (6) preventive behaviour (including medication adherence), (7) affect (affective perceptions, psychological state, stressors, and strains), (8) trust in sources of information, (9) utilisation and assessment of sources of information, (10) trust in authorities/institutions, (11) acceptance of and reactance to the preventive measures, (12) belief that COVID-19 does not exist, (13) coping and resilience, (14) barriers and drivers of getting tested, (15) fairness (acceptance and reactance), (16) satisfaction and well-being, and (17) vaccination intention. Two categories of the ‘WHO Survey Tool and Guidance’ were not adopted (‘Lifting restrictions (pandemic transition phase)’ and ‘Unwanted behaviour’) due to the lack of relevance to the underlying objective. 

Further categories were derived from the literature reviewed such as (18) asthma-related health outcomes, (19) health status, and (20) health care utilisation considering the underlying theoretical framework (Table 1). The item selection for the categories’ subjective and objective HL was informed by the systematic review on HL by Sørensen et al. [3], international and national population-based surveys [2,6,9,10,11,41,53] and research on HL-specific questionnaire development [42,54]. For COVID-19-related HL, the Health Literacy Survey COVID Questionnaire (HLS-COVID-Q22) [2,6], comprising 22 items from four subscales was included. The items refer to a subjective assessment of how ’easy’ or ‘difficult’ it is to find (6 items), understand (6 items), assess (5 items), and apply (5 items) information and are measured by 4-point Likert scales from ‘very easy’ to ‘very difficult’ [2,6]. The HLS-COVID-Q22 instrument has a satisfactory internal consistency (Cronbach’s α = 0.94) [6,7]. 

### 3.2. Quantitative Assessment of Content Validity by CVI Evaluation

Eleven experts participated in the evaluation of the CVI. Seven were female, three were physicians, and eight were researchers in the field of HL, health education, health psychology, or health care. On average, the experts had 10 years of professional experience.

The I-CVI of the first version yielded values between 0.20 and 1.00. Overall, 20 of 386 tested items (without standardised sociodemographic items, n = 12) were considered highly relevant (I-CVI = 1.00). Since sociodemographic items are standardised, established, and validated items, they were not subjected to the quantitative and qualitative content validation process [55]. The S-CVI/Ave was calculated for 19 categories (without the sociodemographic category) and reached values between 0.20 and 0.95. The categories ‘disease pattern’ (S-CVI/Ave = 0.95), ‘health status’ (S-CVI/Ave = 0.87), ‘satisfaction and well-being’ (S-CVI/Ave = 0.80), and ‘trust in authorities/institutions’ (S-CVI/Ave = 0.80) showed acceptable content validity at scale level. The S-CVI/Ave of 14 categories was below 0.80. The item ‘psychological state’ displayed the lowest S-CVI/Ave value (0.20). The overall S-CVI/Ave value for the entire instrument was 0.68, suggesting inadequate content validity at the scale level. The comprehensibility of 200 out of 386 tested items (15 scales) from 15 categories was rated as adequate. Categories such as ‘vulnerability and severity of COVID-19 disease’ (50%), ‘stressors’ (50%), ‘coping and resilience’ (50%), and ‘psychological state’ (40%) had the lowest comprehensibility. Further, low I-CVI values corresponded to low comprehensibility in 129 items (11 scales). The average comprehensibility of the first draft was 74%. As a consequence, 200 single items were removed, and 89 items and two scales were revised. The revision included the following: 76 items were simplified in terms of language, 5 items were rearranged within the instrument, 35 single items (from six categories) were combined to 8 items, one scale was reduced from 10 to 5 items and another from 6 to 3 items.

One hundred and eighty-six items were formed and again evaluated by experts. One hundred and one items were rated as highly relevant (I-CVI = 1.00). The categories ‘coping and resilience’ (S-CVI/Ave = 0.70), ‘vaccination intention’, and ‘psychological state’ (S-CVI/Ave = 0.60) yielded an S-CVI/Ave value below 0.80. The evaluation yielded an S-CVI/Ave of 0.91 and indicated acceptable content validity overall [45]. The second version achieved a comprehensibility of 98% on average. Based on the findings of the second expert rating, 48 items were removed, 21 items and two scales were revised, i.e., 18 items were reworded and linguistically simplified, 2 items were merged to 1 item, two scales (13 and 8 items) were rearranged within the questionnaire, and for 1 item the response options were reduced. The third version consisting of 138 single items was evaluated by cognitive interviews. 

### 3.3. Qualitative Assessment of the Content Validity by Cognitive Interviews 

The sample consisted of nine participants (seven female), with a mean age of 45 years (age range: 20–56 years). All participants had a diagnosis of asthma. The majority of the interviewees stated suffering from allergic asthma (n = 8) and/or exercise-induced asthma (n = 6). 

Content analysis identified six key themes: (1) comprehensibility, (2) relevance, (3) comprehensiveness, (4) reliability of responses, (5) suggestions for improvement, and (6) acceptability. Of the 150 items within the third draft, 12 pertain to sociodemographic variables. The remaining 138 items were examined. Of these, 28 items had been taken unchanged from existing scales and were not elaborated in depth. Comprehension problems occurred in ten items. Fifty-two items referred to unfamiliar or ambiguous terms. Forty-nine items had unclear formulations. Most participants were able to recall discrete experiences and memories without difficulty in order to give a coherent answer. The highest proportion of missing values was observed in two items from question 25 (‘What sources for information do you access to find information about your asthma condition in relation to the pandemic and how reliable do you think these sources are?’) due to the complexity of the answer options. Other factors that were perceived to affect the comprehensibility were unsuitable rating scales due to missing designations of the individual gradations or long and cumbersome phrasing. Forty-two items were rated as ‘difficult to answer’ by at least two participants. In general, the instrument was perceived positive by the interviewees. Eight out of nine participants expressed their willingness to complete the instrument in the context of a population-based survey. The respondents did not show any disinterest or reactance during the interviews. Based on these findings, 22 items were excluded from the third version. Sixteen items were simplified in terms of language, three scales were rearranged within the instrument, missing answer options were added for two items, and for one item and one scale (four items), answer formats were adjusted, two items were combined to one, and within seven scales the number of single items was reduced. Consequently, the final instrument comprises 38 questions (116 single items, including sociodemographic items) within 14 categories measuring COVID-19 pandemic-related HL of individuals with asthma (Appendix A). 

## 4. Discussion

This is a report of the development of a comprehensive HL survey instrument in German for individuals with asthma for use in the context of a pandemic. Based on a theoretical framework and using a mixed-methods approach involving experts as well as participants with asthma, we arrived at an instrument with 116 items in 14 categories. In terms of content, the instrument appears a valid representation of behavioural and psychosocial constructs pertaining to a broad HL understanding and relevant to individuals with asthma during the COVID-19 pandemic.

The instrument’s development and adaptation process was informed by a large body of literature. The structure and the content of the instrument were based on the integrated framework and ‘WHO’s Survey Tool and Guidance’ [43]. An initial draft comprised 398 items (20 categories). The content validity (relevance, comprehensibility, and comprehensiveness) was assessed by expert ratings and target group interviews yielding qualitative and quantitative data.

With regard to relevance, expert evaluation of the first draft attested to a substantial proportion of irrelevant items, particularly within the categories ‘vaccination intention’, ‘psychological state’, and ‘coping and resilience’. Both the HLS-COVID-19 survey [2,6,7] as well as the COSMO-Germany serial surveys [53], suggest, however, that vaccination-related attitudes and psychosocial aspects (e.g., coping strategies, well-being) are important in dealing with the pandemic. We would like to stress that at the time of this study no vaccination was yet widely available. Items were subsequently revised, and redundant items deleted in line with the CVI assessment and the results from the expert ratings and patient interviews. For instance, analyses of the cognitive interviews indicated redundancies among items of the categories ‘asthma-related health outcomes’, ‘knowledge about the coronavirus and COVID-19 disease’, and ‘dealing with pandemic-related health information’. While each category revolves around a distinct theme, some items are considered not to tap into relevant content and are thus redundant. In contrast, ‘affective perception’, ‘vulnerability’, and ‘personal experience with COVID-19′ were considered important by the participants. This is in line with research by Okan et al. [6,7] and Philip et al. [26]. They showed that individuals with chronic diseases including asthma report more worries and fears in the context of the pandemic [6,7,26]. In a similar vein, the COSMO Study Germany demonstrated that social cognitive and affective variables are important in the context of assessing the quality of coping with the coronavirus pandemic in general [53]. 

The average comprehensibility of the revised instrument draft resulted in an increase of 24 percentage points (74% vs. 98%) compared to the first draft. This corresponds with the improved I-CVI values of the second draft, indicating a substantial improvement. The results of the cognitive interviews affirmed the experts’ opinions that, for example, medical terminology (e.g., ‘COPD’, ‘peak-flow meter’) makes comprehension more difficult. Findings also indicated that the terms ‘disease’ and ‘infection’ are generally difficult to distinguish by participants. 

In terms of comprehensiveness, participants expressed that some content-related aspects were missing. (1) Information-seeking behaviour and (2) specific knowledge about individual hygiene measures such as the use of mouth and nose coverings, as well as (3) worry about infection or uncertainty about how to handle one’s asthma during the pandemic, were mentioned as relevant content. The experts, on the other hand, judged the items on the correct handling of mouth and nose coverings as not relevant. We decided to add items on ’information-seeking behaviour’. Further, items pertaining to ‘worries and uncertainty’ were modified (e.g., linguistically simplified, order of answer categories adjusted) and the items of knowledge about use of mouth and nose coverings revised. In terms of negative affect which impacts on health behaviour [42], three aspects were reported: persistent uncertainty about asthma treatment (e.g., drug dosage), unclear COVID-19-related information for individuals with asthma, and an expected stigmatisation of people displaying cold-resembling symptoms (e.g., cough or wheeze). 

Generally, and in terms of COVID-19, it is thought that HL is an indispensable psychosocial factor of health as it shapes the ability to access, for instance, COVID-19-relevant health information, recognise trustworthy sources, understand recommendations for preventive as well as protective measures, and integrate them into personal behaviours and actions [2,4,5,7]. It has been argued that low pandemic-related HL can be considered a risk factor that is associated with significantly lower informedness and reduced occurrence of preventive behaviours [6,7]. In relation to asthma, there is an association between low HL and negative outcomes in terms of disease coping and health-related behaviour such as disease management, treatment adherence, and utilisation of health care services [35,36,37]. This association may be more pronounced during the COVID-19 pandemic [35]. 

### 4.1. Strengths and Limitations

To our knowledge, this is the first study aimed at developing and content-validating a COVID-19-related HL instrument for individuals with asthma. The development was based on a comprehensive review of the available literature, and an integrated framework of HL and survey guidelines. While a balanced approach of experts and people with asthma was chosen to ensure the incorporation of both perspectives in the content validation of the instrument, self-selection bias may have been introduced by voluntary participation. Nevertheless, a wide range of experts were invited to participate in the expert ratings and 11 experts appear a customary size [67]. Similarly, the number of cognitive interviews was nine. Willis (2005) recommends 5 to 15 participants for cognitive interviews [68]. In addition, a stronger emphasis is placed on the assessment of subjective HL as compared to objective HL. Because the instrument is asthma-specific, it cannot be applied to other populations with chronic conditions. 

### 4.2. Implications for Research and Practice

Although the goal of a content-valid questionnaire for the intended population group has been achieved, further validation steps need to be carried out to assess its measurement properties (e.g., reliability, construct validity, sensitivity to change). It may then have the potential to be used in serial cross-sectional surveys to evaluate the COVID-19-related health literacy of individuals with asthma. These surveys could help identify information needs, inadequate asthma management adherence and quality of care provision, inform communication strategies, or psychosocial health impacts. The obtained findings could inform target group-specific interventions aimed at increasing COVID-19-related HL for individuals with asthma. In the meantime, the content-validated instrument provides an appropriate basis for an adaptation to other pandemics or epidemics. In addition, interim application of the questionnaire may be compatible to the HLS-COVID-19 survey and could contribute to a comparison between people with the chronic disease asthma and the general population. Once further validation has been carried out, translation and cultural adaptation into other languages is possible.

## 5. Conclusions

The present article shows that a survey instrument for use in the general population can be adapted to other specific disease populations. It can hence serve as an example for other chronic diseases in which COVID-19 might place a higher risk or burden on the affected population. It allows a modular assessment of HL, its determinants, as well as outcomes and mediators on the pathways between HL and health outcomes. It can eventually be used as a self-administered online instrument (online survey, via e-mail) or as a paper–pencil questionnaire in physicians’ offices. By regular administration, a monitoring of a specific disease population would be possible. The final instrument has 14 categories (116 single items, 17 scales). Repeated administration (monitoring) in individuals with asthma can help identify inadequate levels of HL and its mediators as well as changes over time and unmet needs during the COVID-19 pandemic. However, further validation of this instrument is necessary.

## Figures and Tables

**Table 1:** Content of the asthma-specific survey instrument.

WHO Survey Tool and Guidance [43]	Adapted Survey Instrument							
**Categories**	Categories and Subcategories	Theoretical Framework Level	Item # in Final Version	References	Number of Items in Respective Versions			
					1st	2nd	3rd	Final
**Socio-demography**	Sociodemographic determinants	Sociodemographic determinants	1, 38–48	[55,56]	12	12	12	12
**COVID-19 personal experience**	Personal experiences with COVID-19	Other control variable (experience)	16, 16a	[57,58]	6	4	4	2
**Health literacy (HL)**	COVID-19-related health information management	HL (subjective)	26	[2,7]	22	22	22	22
	Knowledge about COVID-19	HL (objective)	19, 20, 21, 27	[7,57,58,59,60]	54	29	21	16
**COVID-19 risk perception: Probability and severity**	Risk perceptions (vulnerability and severity)	Other control variable (perception)	17, 31	[57]	8	6	6	4
**Preparedness and perceived self-efficacy**	Self-efficacy	Mediator		[57,60]	3	0	0	0
**Prevention—own behaviours**	Health behaviour (COVID-19 and asthma-related)	Health outcome (health behaviour)	30	[61]	53	12	5	3
**Affect**	Affective perceptions	Other control variable (psychosocial impact)	28, 32, 33	[57,60]	37	15	12	8
	Stressors		28, 33		30	3	3	2
	Psychological state				5	7	0	0
**Trust in sources of information**	Dealing with COVID-19-related health information: Trust in sources of information	Other control variable (attitudes)	22, 23	[2,7]	2	2	2	2
**Use of sources of information**	Utilisation and assessment of information sources	Health outcome (health information behaviour)	24, 25, 36, 37	[2,7,57]	30	19	18	16
**Frequency of information**								
**Trust in institutions**	Trust in authorities/institutions	Other control variable (attitudes)		[57,58]	14	0	0	0
**Policies, interventions**	Acceptance of and reactance to the preventive measures	Other control variable (attitudes)		[58]	16	0	0	0
**Conspiracies**	Belief that COVID-19 does not exist	Other control variable (perceptions)	19	[7]	1	1	1	1
**Resilience**	Coping and resilience	Mediator	35	[58]	16	8	5	3
**Testing and tracing**	Barriers and drivers to getting tested	Other control variable (perceptions)	18		7	4	3	3
**Fairness**	Acceptance and reactance	Other control variable (perceptions)		[58]	4	0	0	0
**Lifting restrictions**	Not adopted							
**Unwanted behaviour**	Not adopted							
**Well-being**	Well-being	Other control variable (psychosocial impact)		[58]	6	0	0	0
**COVID-19 vaccine**	Vaccination intention	Other control variable (attitude)		[58]	13	2	0	0
	Additional categories							
	Health care utilisation	Health outcome (health system utilisation)	29, 34	[53,62]	15	12	12	2
	Asthma-related health outcomes	Health outcome (health status)	02–11, 14	[63,64]	17	18	21	17
	General health status	Health outcome (health status)	12, 13, 15	[56,61,65,66]	27	22	3	3
		Total number of items			398	198	150	116

## Appendix A

### Survey Instrument

1. Date of birth
2. Physician diagnosed asthma (yes, no)
3. Age at diagnosis
4. Type of asthma (allergic, non-allergic, asthma with COPD, exercise-induced asthma, do not know, another type)
5. Chronic disease comorbidity (diabetes, bowel, liver, kidney, hypertension, COPD, cancer, another, no other)
6. Medication
7–11. Asthma control test
  - During the last 4 weeks, how much of the time has your asthma kept you from getting as much done at work, school, or home? (all the time, most of the time, some of the time, a little of the time, none of the time)
  - During the last 4 weeks, how often have you had shortness of breath? (more than once a day, once a day, 3 to 6 times a week, once or twice a week, not at all)
  - During the last 4 weeks, how often have your asthma symptoms (wheezing, coughing, shortness of breath, chest tightness or pain) woken you up at night or earlier than usual in the morning? (4 or more nights a week, 2 to 3 nights a week, once a week, once or twice, not at all)
  - During the last 4 weeks, how often have you used your rescue inhaler or nebuliser medication (such as Salbutamol)? (3 or more times per day, once or twice per day, 2 or 3 times per day, once a week or less, not at all)
  - How would you rate your asthma control during the last 4 weeks? (not controlled at all, poorly controlled, somewhat controlled, well controlled, completely controlled)
12. Smoking status and frequency of consumption (cigarettes, cigars, e-cigarettes)
13. Please rate your present health status (very good, good, moderate, poor, very poor)
14. Please indicate which statement you agree with most (cannot answer, do not agree at all, do not agree, agree to some extent, agree)
  - I am well informed about my asthma condition
  - I am familiar with the use of my inhaler and the inhaler medication
  - I am familiar with the use of a peak-flow meter (lung capacity)
  - I know which asthma medication I have to take and why
  - I know the triggers for asthma attacks and exacerbations
  - In order to understand what my doctor tells me I ask questions if necessary
  - Before making decisions about my treatment I critically assess the situation
15. How much does your asthma impair your life? (6-point Likert scale from not at all to very strongly)
16. COVID-19 disease status (symptoms, test status) if negatively tested and/or no symptoms continue with item 16
  - Ever since I had COVID-19 my asthma symptoms have become (less severe, unchanged, somehow more severe, a lot more severe)
17. How harmful or dangerous would a COVID-19 infection be for you? (completely harmless, harmless, rather dangerous, very dangerous, do not know)
18. Attitude towards PCR test (cannot answer, do not agree at all, do not agree, agree to some extent, agree)
  - Getting tested requires a lot of effort and time
  - The PCR test is reliable
  - I would get tested if I had been in contact with a person that tested positive even if I myself had no symptoms
19. The following statements are (correct, incorrect, do not know)
  - COVID-19′s main transmission route is human to human
  - Main symptoms are fever, dry cough as well as loss of smell and taste
  - The coronavirus does not exist
  - The coronavirus can be caught by touching door handles or railings in trams
  - There are drugs for the treatment of COVID-19
20. Prevention measures (yes, no, do not know)
  - Adhere to hygiene rules (e.g., washing one’s hands for 20–30 s)
  - Keep a distance of 1.5–2 m to other people
  - Use the corona alert app
  - Regularly airing indoor areas
  - Wearing a facemask (e.g., when shopping or using public transport)
  - Regular gargling with a mouth rinse
21. How well do you think you are you informed about the coronavirus? (6-point Likert scale from no knowledge at all to a lot of knowledge)
22. About asthma and the coronavirus or the COVID-19 disease I feel (very well informed, well informed, poorly informed, very poorly informed, cannot say)
23. Do you feel confused about coronavirus information? (very confused, somewhat confused, very little confused, cannot say)
24. Compared to the time before the COVID-19 pandemic, these days how often do you seek information about your asthma condition? (less often, unchanged, more often, do not know)
25. What sources for information do you access to find information about your asthma condition in relation to the pandemic and how reliable do you think these sources are? (tick box and then judge reliability (cannot judge, not reliable, rather not reliable, rather reliable, very reliable))
  - Telephone hotlines
  - Health insurance organisations
  - GP
  - Specialist physician
  - Pharmacist
  - Family/Friends/Acquaintances
  - Health-related websites
  - Social media
  - Printed health publications
  - Public broadcasting services
  - Private broadcasting services
  - Other (name)
26. HLS-COVID-Q22: How easy or difficult is it for you to:
  - Find information about the coronavirus on the internet?
  - Find information on the internet about protective behaviours that can help to prevent infection with the coronavirus?
  - Find information in newspapers, magazines, and on TV about behaviours that can help to prevent infection with the coronavirus?
  - Find information on how to recognise if I have likely become infected with the coronavirus?
  - Find information on how to find professional help in case of coronavirus infection?
  - Find information on how I much I am at risk for being infected with the coronavirus?
  - Understand your doctor’s, pharmacist’s, or nurse’s instructions on protective measures against coronavirus infection?
  - Understand recommendations of authorities regarding protective measures against coronavirus infection?
  - Understand advice from family members or friends regarding protective measures against coronavirus infection?
  - Understand information in the media on how to protect myself against coronavirus infection?
  - Understand risks of the coronavirus that I find on the internet?
  - Understand risks of the coronavirus that I find in newspapers, magazines, or on TV?
  - Judge if information on the coronavirus and the coronavirus epidemic in the media is reliable?
  - Judge which behaviours are associated with a higher risk of coronavirus infection?
  - Judge what protective measures you can apply to prevent a coronavirus infection?
  - Judge how much I am at risk for a coronavirus infection?
  - Judge if I have been infected with coronavirus?
  - Decide how you can protect yourself from coronavirus infection based on information in the media?
  - Follow instructions from your doctor or pharmacist regarding how to handle the coronavirus situation?
  - Use information the doctor gives you to decide how to handle an infection with the coronavirus?
  - Use media information to decide how to handle an infection with the coronavirus?
  - To behave in a way to avoid infecting others?
27. Please indicate whether the following statements are correct or incorrect (correct, incorrect, do not know)
  - It is sufficient that the facemask covers only the mouth
  - Medical masks, e.g., FFP-2 masks are more effective than ordinary masks
  - Single use masks have to be disposed of after 8 h or when it has become moist
  - Before putting on the mask and before removal of the mask one should wash one’s hands with soap
28. Please indicate how much you agree with the following statements (cannot answer, do not agree at all, do not agree, agree to some extent, agree)
  - I am confident about how to use a facemask correctly
  - Because of my asthma I find it very difficult to wear a facemask because of breathing difficulty
  - Wearing a facemask protects me sufficiently from infection
29. Since March 2020 have you postponed appointments with your GP or specialist physician because of the pandemic? (yes; no, I attended as scheduled; no, I had no appointments; no, appointment(s) were cancelled)
30. Have there been changes in your asthma treatment since March 2020? (yes, no, do not know)
  - Because of the pandemic, I am more careful in making sure to take my medication regularly
  - Because of the pandemic, I have or intend to interrupt my asthma treatment/medication intake
  - Because of the pandemic, I now take different asthma medication(s)
31. I think that (cannot answer, do not agree at all, do not agree, agree to some extent, agree)
  - I am perfectly able to distinguish my asthma symptoms from those of a COVID-19 infection
  - I am more vulnerable to COVID-19 because of my asthma
  - Should I get COVID-19 the course of the disease could be more severe because of my asthma
32. Please indicate how much you agree with the following statements (cannot answer, do not agree at all, do not agree, agree to some extent, agree)
  - When I cough more, I worry that I might have been infected with COVID-19
  - I worry that my asthma will get worse after a COVID-19 infection
  - I worry about other things (name)
33. The coronavirus is (semantic differential from −3 to +3)
  - I hardly ever think about it–I think about it a lot
  - It makes me feel helpless–I can do something about it
  - Not scary–scary
  - Not stressful–stressful
34. Whose help or advice would you use or have used?
  - Telephone hotlines
  - Health insurance organisations
  - Medical personnel
  - Public health departments
  - Social media
  - Self-help groups
  - Patient societies
  - Counselling services
  - Other (name)
  - I would not use any help or advice
35. What helps you in coming to terms with your asthma condition and the COVID-19 pandemic? (cannot answer, do not agree at all, do not agree, agree to some extent, agree)
  - Talking to friends/family/acquaintances
  - Exercising, going for walks, or other physical activities
  - Other (name)
36. Please indicate how much you agree with the following statement (cannot answer, do not agree at all, do not agree, agree to some extent, agree)
  - There is sufficient information about asthma and the coronavirus available that I find comprehensible and helpful
37. What information would you like about asthma and COVID-19? (text box)
38. Sociodemographic information
